# Ivermectin and COVID-19: Keeping Rigor in Times of Urgency

**DOI:** 10.4269/ajtmh.20-0271

**Published:** 2020-04-16

**Authors:** Carlos Chaccour, Felix Hammann, Santiago Ramón-García, N. Regina Rabinovich

**Affiliations:** 1 ISGlobal, Hospital Clínic - Universitat de Barcelona, Barcelona, Spain;; 2 Ifakara Health Institute, Ifakara, United Republic of Tanzania;; 3 Facultad de Medicina, Universidad de Navarra, Pamplona, Spain;; 4 Department of General Internal Medicine, Clinical Pharmacology and Toxicology, Inselspital, Bern University Hospital, University of Bern, Bern, Switzerland;; 5 Research & Development Agency of Aragon (ARAID) Foundation, Zaragoza, Spain;; 6 Department of Microbiology, Mycobacterial Genetics Group, Preventive Medicine and Public Health, Faculty of Medicine, University of Zaragoza, Zaragoza, Spain;; 7 Harvard T.H. Chan School of Public Health, Boston, Massachusetts

Ivermectin is a widely used drug for the treatment and control of several neglected tropical diseases.^1^ The drug has an excellent safety profile, with more than 2.5 billion doses distributed in the last 30 years, and its potential to reduce malaria transmission by killing mosquitoes is under evaluation in several trials around the world.^2^ Ivermectin inhibits the in vitro replication of some positive, single-stranded RNA viruses, namely, dengue virus (DNV),^3–5^ Zika virus,^4,6^ yellow fever virus,^7,8^ and others.^4,7,9^

Caly et al.^10^ recently reported that ivermectin is a potent inhibitor of the severe acute respiratory syndrome coronavirus 2 (SARS-CoV-2) replication in vitro. Given the coronavirus disease-19 (COVID-19) pandemic, this has understandably resonated widely in the global press.^11^

Caly et al.^10^ report a 5,000-fold reduction in SARS-CoV-2 RNA levels, compared with those in controls, after infected Vero/hSLAM cells were incubated for 48 hours with 5 μM ivermectin. The ivermectin IC_50_ for the virus was calculated at approximately 2.5 μM. These concentrations are the equivalent of 4,370 and 2,190 ng/mL, respectively, notably 50- to 100-fold the peak concentration (*C*_max_) achieved in plasma after the single dose of 200 μg/kg (14 mg in a 70-kg adult) commonly used for the control of onchocerchiasis.^12^ Pharmacokinetic studies in healthy volunteers have suggested that single doses up to 120 mg of ivermectin can be safe and well tolerated.^13^ However, even with this dose, which is 10-fold greater than those approved by the US Food and Drug Administration, the *C*_max_ values reported were ∼250 ng/mL,^13^ one order of magnitude lower than effective in vitro concentrations against SARS-CoV-2.

These findings may seem to discourage follow-up clinical trials with ivermectin. However, some in vivo effect may be possible even if efficacious in vitro concentrations are physiologically unattainable. A recent phase III clinical trial in dengue patients in Thailand, in which a once-daily dose of 400 µg/kg for 3 days was found to be safe but did not produce any clinical benefit,^14^ showed a modest and indirect in vivo effect against DNV.^14^ Previous work by Wagstaff et al.^5^ reported inhibition at much higher in vitro concentrations (25 µM) in DNV-infected Vero cells. Both pharmacokinetic considerations and the relatively long incubation period of DNV might explain the lack of clinical efficacy. Until we have a better understanding of ivermectin’s antiviral mode of action and of appropriate in vitro systems for testing, we caution against using findings in Vero cells as more than a qualitative indicator of potential efficacy.

Very recently, preliminary findings on a potential effect of hydroxychloroquine combined with azithromycin against SARS-CoV-2 were widely publicized,^15^ leading to a surge in demand and self-medication, which resulted in serious harm in some cases and a stock shortage that jeopardized drug availability for other critical conditions for which hydroxychloroquine or chloroquine is the standard of care, that is, vivax malaria, rheumatoid arthritis, and systemic lupus erythematosus. Efficacy claims for hydroxychloroquine against COVID-19 have been questioned in follow-up trials using similar dosing regimens,^16,17^ and we await results of randomized, controlled clinical trials exploring treatment efficacy.

We believe the recent findings regarding ivermectin warrant rapidly implemented controlled clinical trials to assess its efficacy against SARS-CoV-2. These trials may open a new field of research on the potential use of avermectin antiparasitic drugs, including compounds with an improved pharmacokinetic profile, as antivirals.^18^ However, because of the following points, extreme due diligence and regulatory review are needed before testing ivermectin in severe disease.

First, ivermectin, which targets glutamate-gated chlorine channels in invertebrates, may cross-target the GABA-gated chlorine channels present in the mammalian central nervous system (CNS) and cause neurotoxicity.^19^ This is normally prevented by an intact blood–brain barrier (BBB), but in patients with a hyperinflammatory state, endothelial permeability at the BBB may be increased and cause leaking of drugs into the CNS, potentially causing harm.^20,21^

Second, boosted antiretrovirals such as lopinavir/ritonavir and darunavir/cobicistat, which have been widely used against SARS-CoV-2 based on limited evidence, and a number of other drugs, are potent inhibitors of cytochrome P_450_ 3A4, the main metabolic pathway for ivermectin. Concurrent use of these drugs will result in increased systemic exposure to ivermectin. Furthermore, ritonavir and cobicistat can readily inhibit one of the main efflux pumps in the BBB, P-glycoprotein, further favoring neurotoxicity.^22,23^ However, it is encouraging that a recent analysis of ivermectin-related neurotoxic adverse events reported to the WHO Program for International Drug Monitoring found only one case of 1,668 reports in which concomitant use of antivirals was associated with neurotoxicity.^24^

Third, as earlier, available evidence suggests that levels of ivermectin with meaningful activity against SARS-CoV-2 would not be achieved without extraordinary, potentially toxic increases in ivermectin dosing levels in humans. However, evidence from animal models showing up to 3-fold higher levels in pulmonary tissue than in plasma 1 week after oral dosing leaves the door open for further research, in particular for the treatment of respiratory viruses.^25,26^

The discovery of ivermectin’s activity against SARS-CoV-2 gives reason for hope, but off-label and compassionate use requires careful risk–benefit considerations,^27^ especially in critically ill patients. A path to consider is evaluation first of impacts on virologic outcomes in uncomplicated, low-risk patients early in the course of the disease. Well-conducted clinical trials informed by robust pharmacokinetic models should be considered to validate the impact before the use of ivermectin to treat SARS-CoV-2 is implemented.
